# Oral microbiota and metabolites: key players in oral health and disorder, and microbiota-based therapies

**DOI:** 10.3389/fmicb.2024.1431785

**Published:** 2024-08-20

**Authors:** Narjess Bostanghadiri, Mobina Kouhzad, Elahe Taki, Zahra Elahi, Amin Khoshbayan, Tahereh Navidifar, Davood Darban-Sarokhalil

**Affiliations:** ^1^Department of Microbiology, School of Medicine, Iran University of Medical Sciences, Tehran, Iran; ^2^Department of Genetics, Faculty of Science, Islamic Azad University North Tehran Branch, Tehran, Iran; ^3^Department of Microbiology, School of Medicine, Kermanshah University of Medical Science, Kermanshah, Iran; ^4^Department of Basic Sciences, Shoushtar Faculty of Medical Sciences, Shoushtar, Iran

**Keywords:** oral microbiota, bacteria, periodontal disease, oral cavity, microbiota metabolites

## Abstract

The review aimed to investigate the diversity of oral microbiota and its influencing factors, as well as the association of oral microbiota with oral health and the possible effects of dysbiosis and oral disorder. The oral cavity harbors a substantial microbial burden, which is particularly notable compared to other organs within the human body. In usual situations, the microbiota exists in a state of equilibrium; however, when this balance is disturbed, a multitude of complications arise. Dental caries, a prevalent issue in the oral cavity, is primarily caused by the colonization and activity of bacteria, particularly streptococci. Furthermore, this environment also houses other pathogenic bacteria that are associated with the onset of gingival, periapical, and periodontal diseases, as well as oral cancer. Various strategies have been employed to prevent, control, and treat these disorders. Recently, techniques utilizing microbiota, like probiotics, microbiota transplantation, and the replacement of oral pathogens, have caught the eye. This extensive examination seeks to offer a general view of the oral microbiota and their metabolites concerning oral health and disease, and also the resilience of the microbiota, and the techniques used for the prevention, control, and treatment of disorders in this specific area.

## 1 Introduction

**Figure 1:**
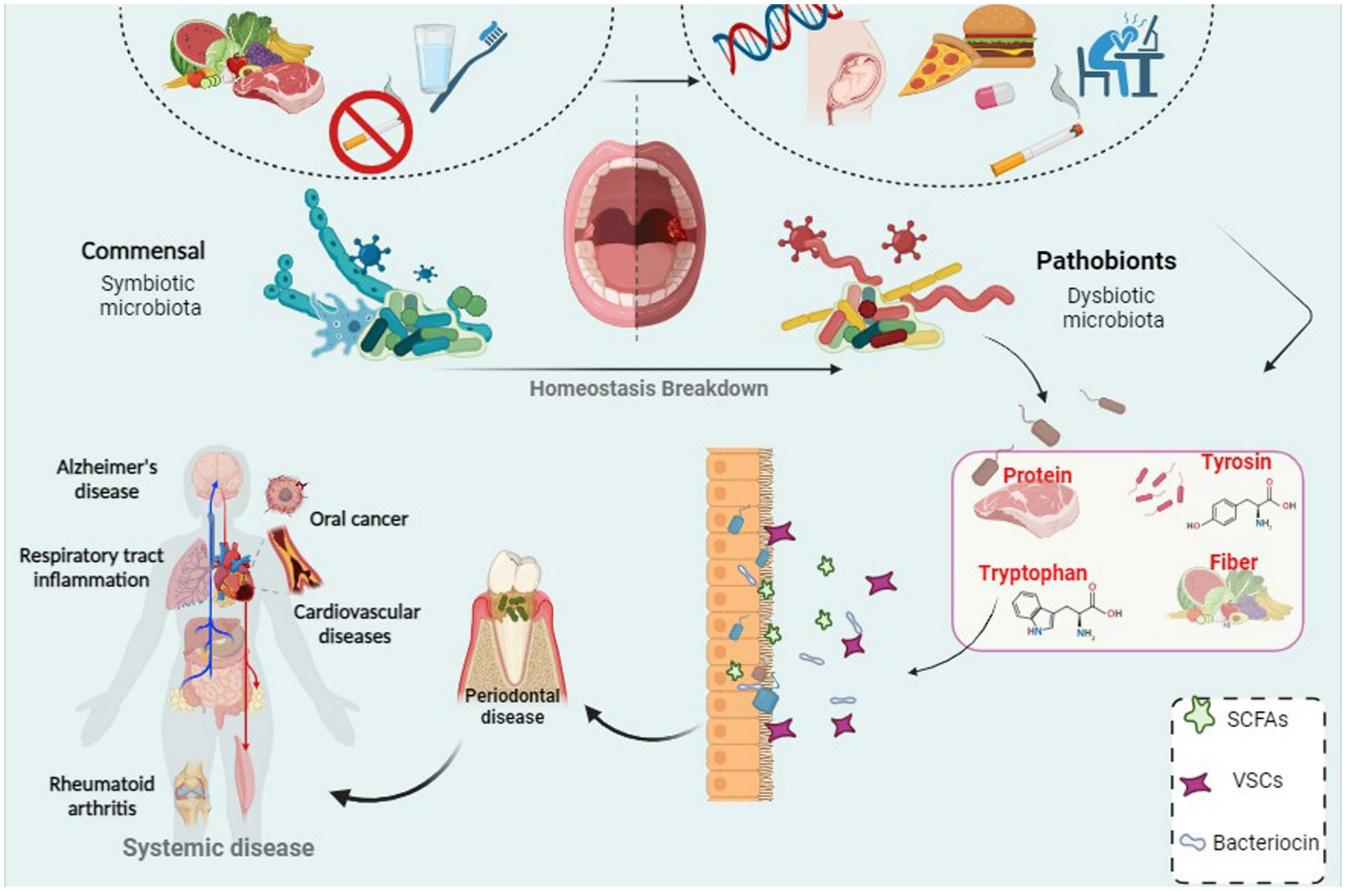
A correlation between bacterial infection and periodontal disease which can led to systemic disease and cancer.

In the 1700s, Antonie van Leeuwenhoek made a groundbreaking discovery while studying dental plaque under the microscope. The discovery of bacteria in dental plaque is one of the first areas of study in traditional microbiology (Mark Welch et al., 2016; Bowen et al., 2018; Lamont et al., 2018). The oral cavity is home to a rich population of microorganisms, many of which are unique and evolved specifically for oral colonization (Mark Welch et al., 2016; Lamont et al., 2018). The oral microbiota is the second most important and the richest microbial community after the gut and is one of the five research priorities of the Human Microbiome Project. Over 1,100 different taxonomic groups are recorded in the Human Oral Microbiome Database, and the genera *Streptococcus*, *Neisseria*, *Veillonella*, and *Actinomyces* are associated with the core microbiome (Bäckhed et al., 2012; Wade, 2013; Franzosa et al., 2015; Bowen et al., 2018; Deo and Deshmukh, 2019; Herremans et al., 2022). Among them, facultative anaerobic bacteria such as *Streptococcus* and *Actinomyces* predominate in the oral cavity. The low oxygen tension in the subgingival area creates favorable conditions for an increasing number of strict anaerobes such as *Bacteroidaceae* spp. and *Spirochaetes*. Identification of microbial communities has a major role in physical and metabolic exchanges between species, which can be cooperative or antagonistic (Mark Welch et al., 2016; Bowen et al., 2018; Lamont et al., 2018). Since 2000, with the introduction of cost-effective genetic sequencing, scientists have focused on the diversity of oral microbial communities and understanding their impact on systemic diseases (Bäckhed et al., 2012). Using modern sequencing technology and next-generation sequencing (NGS) systems, the barriers of traditional culture-based techniques can now be overcome (Franzosa et al., 2015). The microbial community in the body has various functions and is crucial for host health. However, we only become aware of them when the microbiota is out of balance and disease occurs (Zaura et al., 2009; Deo and Deshmukh, 2019). Moreover, recent evidence supports a link between systemic disease and the oral microbiome. The potential for many oral microbes to interplay with an inflammatory microenvironment may be related to this phenomenon. In addition, poor oral hygiene is closely associated with adverse systemic health, and research on oral health and related factors has become a subject of attention (Caselli et al., 2020; Lippi and Mattiuzzi, 2020). Therefore, in the present review, we investigated the diversity of oral microbiota and its influencing factors, and also investigated the association of oral microbiota with oral health and the possible effects of dysbiosis and oral disorder (Figure 1).

## 2 Composition and diversity of the oral microbiota

The oral cavity is a complex habitat that allows microorganisms to interact with the host, and is continuously subjected to both inhaled and ingested microorganisms. Of the more than 700 species of viruses, bacteria, fungi, and protozoa in this microbial community, only 54% have been identified and can be cultured. Another 14% of these species are cultivable but have not so far been recognized, and the other 32% have not been cultivated at all (Bäckhed et al., 2012; Wade, 2013; Herremans et al., 2022). It is essential to notice that everyone has their own unique “microbial identity” consisting of their microbial community. The diversity of the human oral microbiome is influenced by the microhabitat. However, in general terms, the human oral microbiome can be defined as having two major but variable components (Franzosa et al., 2015; Deo and Deshmukh, 2019). The core microbiome is similar among healthy individuals (Zaura et al., 2009), while environmental factors and physiological changes specifically influence the diversity of the microbiome (Lippi and Mattiuzzi, 2020). As a result of both genetic and environmental factors, the composition of the oral microbial community can change over time (Caselli et al., 2020; Herremans et al., 2022). Based on the results of NGS, it has been shown that most of the bacteria in the mouth belong to the phyla *Proteobacteria*, *Firmicutes*, *Bacteroidetes*, *Oriarchyota*, *Fusobacteria*, *Tenricotes*, *Actinobacteria*, and *Spirochaetes* (Dewhirst et al., 2010). *Chloroflexi*, TM7, GN02, *Synergistetes*, *Chlorobi*, SR1, and WPS-2 are among the lesser known potential phyla in the mouth (Camanocha and Dewhirst, 2014). The mouth is a complex environment that contains diverse microbial populations and undergoes significant changes during life stages (Sedghi et al., 2021). During the menstrual cycle, physiological hormones cause changes in bacteria such as *Oribacterium* spp., *Campylobacter* spp., *Haemophilus* spp., and *Prevotella* spp. (Bostanci et al., 2021). Another study found that the prevalence of oral *Neisseria* spp., *Porphyromonas* spp., and *Treponema* spp. was higher in pregnant women than in non-pregnant women, but *Streptococcus* spp. and *Veillonella* spp. were more common in non-pregnant women than in pregnant women (Saadaoui et al., 2021). These findings assume that the oral microbial ecosystem should be analyzed with respect to the age of the host and its particular biological niche within the oral cavity, as it is continuously exposed to external chemicals (Jiang et al., 2015; Baker and Edlund, 2018; Oliveira et al., 2021). Oral archaea in the oral microbiome are less diverse and less abundant than bacteria. Initially, it was assumed that they were only methanogenic, but several studies have also found non-methanogenic bacteria in the oral cavity (Li et al., 2009; Wade, 2013; Dame-Teixeira et al., 2020). Although not pathogenic, these microorganisms have been identified in inflamed pulp tissue, subgingival biofilms, and caries biofilm samples. Further research is required to determine their potential pathogenicity (Efenberger et al., 2015; Aleksandrowicz et al., 2020; Dame-Teixeira et al., 2020).

There has been very little research on the mycobiome, which refers to the fungal and other microbes present in the oral cavity. However, up to 101 species of fungi have been identified as part of the healthy oral microbiota, including *Cryptococcus* spp., *Aureobasidium* spp., *Aspergillus* spp., *Cladosporium* spp., *Saccharomyces* spp., *Candida* spp., *Fusarium* spp., and Aspergillus spp. (Arboleya et al., 2015; Fujiwara et al., 2017; Godlewska et al., 2017; Gomez-Arango et al., 2017; Grant et al., 2019). *Anelloviridae*, *Herpesviridae*, and *Papillomaviridae* are the most common oral virome (Holmes et al., 1998; Welch et al., 2020).

## 3 Factors influencing composition and diversity of oral microbiota

Various internal and external factors, as well as lifespan, can affect oral health. The concept of resilience refers to a system’s capability to quickly recover its equilibrium state after a perturbation (Rosier et al., 2020). Thus, in the interplay between host and oral microbiota, resilience plays a vital contribution to the maintenance of health. Similarly, microbiota resilience could minimize the impact of perturbation while maintaining the symbiotic state, thereby inhibiting dysbiosis and preventing bacterial disease (Liu et al., 2012; Nascimento, 2018). Conversely, Bacteria maintain their balance to maintain oral health. The adaptability of the human oral cavity is affected by host conditions such as genetics, age, immune system, lifestyle and oral environment such as diet, pH, gingival crevicular fluid, and saliva. These factors can induce alterations in the diversity of the community of oral microbes (Chattopadhyay et al., 2019; Nascimento et al., 2019).

### 3.1 Internal factors

#### 3.1.1 Host genetics

Modern genomic techniques have demonstrated the impact of host genetic factors on the diversity of the oral microbiota community (Heilbronner et al., 2021). However, reports on the heritability of the oral microbiota are conflicting (Wescombe et al., 2012; Bushin et al., 2020).

For example, Esberg et al. highlighted the effect of genetic factors on the composition of the oral microbiota through various factors such as immune pathways in 836 Swedish twins. By studying separate groups of adolescents and adults using 16S rRNA gene sequencing, this research indicated that genetic factors affect the host’s immunity, the composition of oral microbiota species, and susceptibility to tooth disorders such as periodontitis and caries (Ferreira et al., 2014). Studies conducted on twins have proven that hereditary traits have a significant contribution in the composition of oral microbiota. Monozygotic twins had a much more similar oral microbial profile than dizygotic twins, highlighting the importance of host genetics (Lin et al., 2021; Zhang J. S. et al., 2022). In addition, research has shown that the abundance of cariogenic species in plaque and saliva is highly heritable trait (Zhang et al., 2018).

In a study of oral metagenomic analysis of over 1,915 cases, human genetics is responsible for a minimum 10% of the oral microbiome composition in all people. These findings suggest that the oral microbiome persists or recurs in people primarily because of changes in their genetics. It also appears that certain host miRNAs may regulate the growth of specific oral bacteria, which may help predict the risk of dental problems such as gum bleeding and plaque (Duran-Pinedo, 2021). A study conducted in 2021 analyzed the oral microbiota of adoptive mother–child pairs and concluded that acquired factors, such as contact and shared environment, are instrumental in altering and transferring oral microbiota from host genetics. However, because of inconsistent findings, more research is necessary to provide a better insight into the relationship between host genetics and the oral microbiome (Nowicki et al., 2018).

#### 3.1.2 Saliva

The first stage of food digestion occurs in the oral cavity, where chewing, facilitated by the teeth, and subsequent swallowing occurs. Saliva contains peptides, vitamins, amino acids, glycoproteins, and proteins. As a result, the oral microbiota is enriched with saliva-derived nutrients for growth and development. In the context of cleaned teeth, salivary biomolecules form an “acquired pellicle” on the tooth surface by depositing a thin layer containing proline-rich proteins, statherin and histatins, which play a crucial role in oral health (Hannig et al., 2017; Hu et al., 2023). In addition, gingival crevicular fluid (GCF) provides a source of nutrition for the microbiota, containing glycoprotein, heme and albumin (Cornejo Ulloa et al., 2019; Wade, 2021). In the absence of compounds and immune properties of saliva, non-oral bacteria and fungi can colonize and multiply in a dry oral cavity (Dabdoub et al., 2016; Tuominen and Rautava, 2021). The buffering system of saliva contains bicarbonate and phosphate, which are essential for the maintenance of the pH value of the mouth at a neutral level, which is optimal for bacterial growth. The sugars in saliva are rapidly metabolized by the oral microbiota, resulting in the production of acidic components that significantly lower the pH of saliva. This acidic environment, facilitates irreversible tooth decay by activating the saccharolytic activity of bacteria such as *Streptoccucs mutans*, *Actinomyces* spp., *Bifidobacteria* spp., *Propionibacterium acidifaciens* and *Lactobacilli*, which convert sugars into acidic products. Under these circumstances, microbial diversity is reduced, leading to the formation of cariogenic microbiota, including acid-tolerant and saccharolytic bacteria (Rosier et al., 2018; Cornejo Ulloa et al., 2019).

### 3.2 External factors

#### 3.2.1 Indoor environment

The indoor microbiome and its metabolites, such as flavonoids and mycotoxins, play a significant role in shaping the oral microbiome’s composition and function. The indoor environment, where individuals spend a substantial amount of time, hosts diverse microbial communities that can influence the human microbiota, including the oral microbiome. Studies suggest that the diversity and composition of indoor microbiomes can impact the microbial populations in the human body. A healthy indoor microbiome can introduce beneficial bacteria into the oral cavity to support oral health. Conversely, an indoor environment with a high prevalence of pathogenic microbes can disrupt the oral microbial balance, leading to conditions such as periodontal disease and dental caries. Effective management of the indoor environment through proper ventilation and regular cleaning is crucial to mitigate the negative impact on the oral microbiome (Adams et al., 2015; Zhang et al., 2023).

Flavonoids are plant polyphenols found in various foods and beverages, known for their antimicrobial and anti-inflammatory properties. These compounds significantly influence the oral microbiome by inhibiting the growth of harmful bacteria and promoting beneficial species. For instance, catechins in green tea exhibit antibacterial activity against *Streptococcus mutans*, a major contributor to dental caries (Ferrazzano et al., 2009). Flavonoids also reduce oxidative stress and inflammation in the oral cavity, thereby supporting a healthy and balanced oral microbiome (Meena et al., 2020). Mycotoxins are toxic metabolites produced by fungi and are commonly found in contaminated food and indoor environments. Exposure to mycotoxins, such as aflatoxins and ochratoxins, can adversely affect the oral microbiome. Mycotoxins disrupt the balance of oral microbial communities by selectively inhibiting beneficial bacteria and allowing pathogenic fungi and bacteria to proliferate. This disruption increases the risk of oral infections and inflammation, which can lead to systemic health problems. In addition, mycotoxins can impair the immune response, further compromising the resilience of the oral microbiome to pathogens (Bryden, 2012; Bai et al., 2021).

#### 3.2.2 Diet and nutrition

Understanding the impact of diet on oral microbes is essential to developing successful oral disease prevention strategies (Yang et al., 2018). Since the 1960s, there has been a shift in dietary patterns toward a Westernized style. This change is characterized by the consumption of certain foods such as meat from farmed animals, high-sugar dairy products, refined vegetable oils, and processed grains. These diets have been in associated with pathological modifications in the oral microbiota, including an increase in the proportion of acid-producing and acid-tolerant organisms (Karpiński, 2019; Sun et al., 2020). Dietary variables significantly influence the oral microbiota biofilm, which may disrupt the homeostasis of specific bacterial species (Yang et al., 2018). Evidence suggests that macro- and micronutrients can modify pro- and anti-inflammatory cascades and influence the inflammatory state of an individual at rest. The oral microbiota is nourished by the diet, which also causes selective pressures that favor the growth and proliferation of organisms that are best suited to utilize certain food resources provided by the host (Lee et al., 2017).

An oral health study evaluated the effect of traditional diets on the dental health of “bush dwellers” and “village dwellers” who were consuming an increasingly externalized diet. The results supported the idea that hunter-gatherer societies have better dental health than agricultural societies. Women who resided in villages and ate a diet rich in agricultural products had a higher prevalence of tooth decay and periodontal disease than those who lived in the bush and consumed a diet rich in wild foods such as legumes. However, cultural factors such as excessive honey consumption and smoking affect the dental health of bush dwelling men, who do not maintain the same level of dental health as their female counterparts (Zhang L. et al., 2020b). In addition to the well-established association between simple carbohydrate consumption and dental caries, several dietary factors may help prevent dental caries. The results of an *in vivo* study revealed that frequent usage of hard candy increased the rate of oral streptococci such as *Streptococcus parasanguinis*, *Streptococcus gordonii*, and *Streptococcus sanguinis* while generally decreasing some species of the oral microbiota such as *Haemophilus* spp. and members of the *Proteobacteria* phylum (Abranches et al., 2018; Hampelska et al., 2020).

Recent research has shown that the entero-salivary nitrate-nitrite-nitric oxide pathway, which promotes nitric oxide (NO) maintenance, is a favorable symbiotic connection between oral microbiota and the human host. Here, nitrate concentrated in the salivary glands is converted to nitrite by facultative anaerobic oral bacteria, which is subsequently ingested and absorbed into the circulation before being further converted to NO. Moreover, the daily use of antiseptic mouthwash for up to 7 days can disrupt the oral microbiome of healthy non-athletes, resulting in a decrease in plasma and oral nitrite levels and elevated blood pressure without dietary intervention. This is an indication of the essential contribution of the oral microbiome to this condition. These findings suggest which nutrition can alter the oral microbiome that affects the entero-salivary pathway and NO homeostasis, but further research is needed to explore this possibility in depth (Vinasco et al., 2019; Wu et al., 2022).

#### 3.2.3 Smoking

The oral area represents one of the first sites on the body to be subjected to cigarette smoke, leading to increased carcinogenesis, reduced mucosal immunity, and altered oral microbiota (Al Bataineh et al., 2020). Numerous toxicants from cigarette smoke can disrupt the oral cavity’s microbial diversity by antibiotic effects, oxygen reduction, or another way (Wu et al., 2016). Smoking with alternation of oxygen levels can favor the survival of microaerophilic bacteria over commensal beneficial species (Jaspers, 2014). It also promotes the development of biofilm and increases the adherence of pathogens, such as *Streptococcus pneumoniae*, *Staphylococcus aureus*, and *S. mutans*, to the epithelial layer. According to some evidence, former smokers’ oral microbiota was more similar to that of current smokers than non-smokers, suggesting smoking effects on oral microbiota may last for years. The oral cavity of smokers tends to be more colonized with disease-associated bacteria such as *Actinomyces*, which have been associated with the occurrence of dental diseases such as periodontitis and caries. Additionally, *Actinomyces* spp. in the salivary microbiota have also been linked to the development of liver cancer (Li et al., 2020; Jia et al., 2021). Wu et al. found that current smokers had a reduced frequency of aerobic metabolic pathways, such as tricarboxylic acid (TCA) cycle and oxidative phosphorylation, and higher frequencies of glycolysis and other oxygen-independent carbohydrate metabolism pathways. Non-smokers had more Proteobacteria than current smokers, while the difference between former smokers and never smokers was not significant. *Atopobium* spp. and *Streptococcus* spp. were enriched relative to never smokers, but the genera *Capnocytophaga* spp., *Peptostreptococcus* spp. and *Leptotrichia* spp. were reduced. The study also found that non-proteobacterial taxa was associated with smoking. Overall, evidence shows that cigarette smoke creates a setting that is more conducive to facultative aerobics than robust aerobics (Jia et al., 2021). On the other hand, cigarette smoking affects the respiratory immune system at several levels (Tuominen and Rautava, *2021*), which resulted in the induction of inflammatory cytokines such as IL-8, TNF-α, and MCP-1, due to the stimulation of innate immune and inflammatory cells such as neutrophils and macrophages (Jaspers, 2014).

#### 3.2.4 Pregnancy

It is vital to maintain good oral hygiene during pregnancy because the mother’s oral hygiene has a direct effect on the child’s oral hygiene. Hence, the elimination of maternal oral infections during pregnancy is critical, as it can prevent or delay colonization of the infant’s oral cavity with oral pathogens (Jang et al., 2021). Altered oral microbiota during pregnancy may have implications for maternal oral health, birth outcomes, and infant oral health because a stable microbiota helps maintain stable oral and general health. Therefore, it is critical to understand changes in the oral microbiota during pregnancy, their relationship to maternal health, and their impact on birth outcomes (Jang et al., 2021). Current studies have revealed that the diversity of oral microbiota in pregnant women remains stable throughout pregnancy. Although, the diversity of the oral microbiota changes in a pathogenic manner and then returns to a normal or healthy microbiome after delivery. It is believed that these changes are caused by female hormones such as progesterone and estrogen (Ye and Kapila, 2021). Previous reports have shown a high frequency of *Prevotella intermedia sensu lato* over the second trimester. Subsequent studies have found that the growth of certain bacterial species, such as *Porphyromonas gingivalis* has been found to have elevated levels of progesterone and estrogen, which are part of the fumarate reductase system of the pathogen (Lin W. et al., 2018). A 2015 study by Fujiwara et al. found that the number of pathogenic bacteria, such as *P. gingivalis* and *Aggregatibacter actinomycetemcomitans*, in gingival crevices increased in pregnant women during the early and middle stages of pregnancy compared to non-pregnant women (Fujiwara et al., 2017).

## 4 Role of the oral microbiota in oral health

The oral cavity is a warm, moist, and nutrient-rich environment that provides a suitable niche for the colonization of microbiota. Early colonization is exhibited by Gram-positive bacteria, such as *Streptococci* (*S. sanguinis*, *S. mutans*, *S. sobrinus*, *S. gordonii*, and *S. oralis*), as well as *Actinomyces*, through adhesion to various surfaces, including gingival surfaces and teeth covered by pellicles (O’Mullane et al., 2016; Thurnheer and Belibasakis, 2018).

The oral microbiota strives to retain a healthy dynamic state and a symbiotic relationship in the complex oral ecosystem, which is referred to as eubiosis. In addition, these bacteria can self-aggregate and co-aggregate with other bacterial species, including some Gram-negative bacteria, through various bacterial glycoproteins and polysaccharides. This process serves as the basis for biofilm formation (Holmes et al., 1998; Bürgers et al., 2010). However, this state of eubiosis can be interrupted and transformed into a state of dysbiosis (Radaic and Kapila, 2021). Dysbiosis is characterized by the involvement of bacterial metabolites in periodontal disease, as well as a marked difference in the metabolic profiles of the oral microbiota when compared with healthy conditions (Heilbronner et al., 2021). In this situation, oral microbiota may be associated with the pathological processes of several diseases, such as inflammatory bowel disease (IBD), arthritis, colorectal and pancreatic cancer, and Alzheimer’s disease, by serving as a reservoir for opportunistic pathogens.

## 5 Role of oral microbiota metabolites in health

The oral bacterial population produces a significant source of metabolites in the mouth which are associated with critical processes in human health or disease. Bacterial metabolites include by-products of carbohydrate, lipid, and protein metabolism such as short chain fatty acids (SCFAs), amines, and gasses (Mai et al., 2017) (Table 1). These metabolites are used by the medical and biotechnology industries as antimicrobial, antifungal, and anticancer agents. Saliva contains glycoproteins and amino acids that are broken down into their subunits by oral microbiota and human enzymes. Under anaerobic conditions, acid-producing bacteria including, *Actinomyces* spp., *Streptococcus* spp., and *Lactobacillus* spp. metabolize deuterated sugars to ethanol, lactic acid, formic acid, and acetic acid via the Embden-Meyerhof-Parnas pathway (Takahashi, 2015; Rosier et al., 2020). The nitrate-reducing bacteria use the acids that are produced as a source of carbon. Then, they raise the pH with ammonia from arginine and urea metabolism, neutralizing the acid and creating a balanced oral ecosystem. In this mechanism, nitrate can be reduced to ammonia (NH3) by the bacterial nitrate-reducing ammonium (DNRA) pathway, raising the pH (Efenberger et al., 2015). As a result, the simultaneous presence of acid-producing and nitrate-reducing bacteria helps maintain a neutral oral cavity (Kistler et al., 2013; Abusleme et al., 2021).

**Table 1:** Oral microbiota, their metabolites, and functions.

Metabolites	Bacteria	Function in oral health	Function in oral disorder
SCFAs	*Streptococcus* *Lactobacillus* *F. nucleatum* *T. forsythia* *P. intermedia* *P. gingivalis* *T. denticola*	Regulating the immune equilibrium between the host defense system and the maintenance of commensal immune tolerance (Jiao et al., 2020)	Increasing the pathogenicity of another oral microbiota Reconstruction of the biofilm species Developing the SCFAs producing bacteria Inducing tissue inflammation in subgingival and supragingival Reducing the expression of ICAM-1 and the transmigration of immune cells Reduction the production of mediators and phagocytosis of neutrophils Reactivation of latent viruses (Roslund et al., 2021)
VSCs	*P. gingivalis* *P. intermedia* *P. nigrescence* *T. forsythia*	Promoting the antitumor immune responses Suppressing the H2S-mediated proliferative capacity on oral cancer cells	Stimulation of the inflammatory cytokines IL-1*β* and IL-18 Promoting the inflammatory response Oral halitosis (Feng et al., 2023)
Bacteriocin	*Streptococcus* *Lactobacillus* *Fusobacterium* *Prevotella*	Probiotic effects Anticancer effects	Interfering with the growth of others microbial competitor Improving the colonization and establishment of bacteria in oral biofilms (Barbour et al., 2022)
Organic acid	*Lactobacillus*, *Bifidobacterium*, *Saccharomyces*, *Enterococcus*, *Bifidobacterium Streptococcus*, *Pediococcus*, *Leuconostoc*	Antagonistic activities against adverse microorganisms (Zhang G. et al., 2020)	Affecting the ecology of the oral microbiome and demineralization of enamel surface (Saha et al., 2023)
Nitrate	*Rothia* *Neisseria* *Porphyromonas* *Actinomyces* *Granulicatella* *Veillonella* *Prevotella* *Haemophilus* *Fusobacterium* *Brevibacillu* *Kingella*	Maintaining the pH of oral cavity in normal range Reducing lactic acid accumulation Controlling the development of caries	It increases in response to as a response of salivary glands to the inflammatory process (Sánchez et al., 2014)

SCFAs, Short chain fatty acids; VSCs, volatile sulfur compounds.

Lugdunin is an example of an oral microbiota metabolite. It is synthesized by *Staphylococcus lugdunensis*, a commensal bacterium found in the mouth. It belongs to thiazolidine-containing cyclic peptides and has been identified as a potent inhibitor of methicillin-resistant *S. aureus*, a superbug that poses a significant threat to human health. *S. gordonii* and *Actinomyces* spp. can metabolize basic amino acids such as arginine to produce ornithine, and citrulline and release other by-products including ammonia, ATP and CO_2_ by a number of reactions referred to as the arginine deiminase system (ADS) (Liu et al., 2012). The alkalinity created by this process can eliminate the acidification produced by sugar metabolism. In fact, some findings showed that lower arginine catabolic capacity, as determined by measuring ADS activity, had correlated with the formation of carious lesions in children. This event is the result of ammonia production by ADS, which leads to the elimination of glycolytic demineralizing acids, thereby promoting the development of a microbiota that protects dental health (Nascimento, 2018; Nascimento et al., 2019).

Therefore, using ADS+ strains as probiotics and adding arginine to oral health products can be a helpful strategy for preventing and treating dental caries (Nascimento et al., 2019). Oral alkaline producing species such as *S. gordonii* and *Actinomyces* spp. can be used to neutralize the acidity of the oral cavity in oral cancer and limit the development of acidogenic bacteria (Chattopadhyay et al., 2019). Also, the oral microbiota produces several classes of metabolites, such as bacteriocins, lantipeptides (or lantibiotics) and sactipeptides that are classified as Ribosomally synthesized and post-translationally modified peptides (RiPPs). They can integrate into the target cell membrane and induce membrane perforation, which is their main mode of action. Because of their cell-killing activity, these peptides have been indicated for use in the treatment of cancer (Barbour et al., 2022).

The most extensively studied examples of RiPPs are bacteriocins, which are synthesized by some bacteria. They are typically described as antimicrobial peptides or proteins with the ability to inhibit or kill specific pathogens. The most widely studied oral microbial clusters that produce bacteriocins are members of *Prevotella* spp., *Streptococcus* spp., *Fusobacterium* spp., and *Lactobacillus* spp., which secrete one or more bacteriocin-like inhibitory factors (Heilbronner et al., 2021). On the other hand, it has been shown that lantibiotics, as one of the categories of bacteriocins, interfere with the membrane potential of target cells. An example of lantibiotics synthesized by oral community microbiota is salivaricin A. This is the primary lantibiotic produced by *S. salivarius* (Wescombe et al., 2012). Sactipeptides are a type of RiPP that possess a sactionine thioether bridge that is post-translationally incorporated and required for their ability to exert antimicrobial activity. Streptosactin is an example of a sactipeptide synthesized by *Streptococcus thermophilus* and oral microbiota, such as *Streptococcus constellatus*, *S. gordonii*, *S. oralis*, and *S. parasanguini* (Bushin et al., 2020).

## 6 Imbalance of oral microbiota in oral disorders

An imbalance between the microbiota and the host caused by certain factors can lead to diversity in the microbial community. This can cause to oral and dental diseases in which beneficial bacteria are destroyed and pathogens proliferate. Under these conditions, the immune system is unable to fight the pathogens, exposing the host to tissue and dental damage such as dental caries, periodontal disease, and carcinogenic metabolites that can cause cancer and other systemic diseases (Verma et al., 2018; Radaic and Kapila, 2021). In addition, it has also been contributed to various types of malignancies, including gastric, colorectal, liver, lung, esophageal, and breast malignancies (Takahashi, 2015). In this section, we focus on some diseases that result from perturbations in oral microbiota.

### 6.1 Dental caries (cavities)

In general, investigation of the oral microbiota composition in caries revealed that the bacterial population in caries is less diverse than in the healthy state (Zhang Y. et al., 2022). Although, the genera *Streptococcus* spp. and *Lactobacillus* spp., have been studied as the most common causative agents of dental caries, current evidence using meta transcriptomics approaches has revealed a broad spectrum of microbiota contributing to dental caries (Lin et al., 2021).

Moreover, *Actinomyces* spp., *Fusobacterium* spp., *Porphyromonas* spp., *Rothia* spp., *Granulicatella* spp., *Gemella* spp., *Selenomonas* spp., *Bifidobacteria* spp., *Scardovia* spp., *Corynebacterium* spp., *Granulicatella* spp., *Propionibacterium* spp., *Bifidobacterium* spp., and *Scardovia* spp. are strongly related to caries development (Zhang et al., 2018; Duran-Pinedo, 2021; Zhang J. S. et al., 2022). These microbiota interact with each other in a dynamic polymicrobial biofilm and induce the progression of caries from early-onset (primary demineralization) to deeper damage with dentin exposure (Zhang et al., 2018).

### 6.2 Periodontal diseases (gingivitis and periodontitis)

Chronic inflammation caused by oral microbiota can damage the structures that support the teeth, leading to gingivitis and periodontal disease (Duran-Pinedo, 2021). Gingivitis is a reversible stage that begins at the bottom of the apical gingival border and modifies the supra and subgingival sulci (Radaic and Kapila, 2021).

Molecular analyses of bacterial communities have demonstrated that a distinct cohort of oral microbiota, including the genera *Oribacterium* spp., *Leptotrichia* spp., *Tannerella* spp., *Lachnospiraceae* spp., *Lachnoanaerobaculum* spp., *Lautropia* spp. as well as *Prevotella oulorum* and *Fusobacterium nucleatum* are more abundant during gingivitis (Kistler et al., 2013; Nowicki et al., 2018). The rapid development of gingivitis leads to the accumulation of oral microorganisms in the gingival crevices, resulting in inflammation and ultimately the destruction of the periodontium and alveolar bone (Sedghi et al., 2021). Imbalance of oral microbiota is a consequence of periodontitis, leading to a shift from commensal to pathogenic species (Radaic and Kapila, 2021).

Examination of the oral microbiota of healthy individuals and those with periodontitis revealed that *Actinomyces*, *Rothia*, and *Streptococcus* spp. are predominant in the healthy oral cavity compared with periodontitis, whereas periodontitis was associated with a greater diversity of pathogenic taxa including *Treponema* spp., *P. gingivalis*, *Porphyromonas endodontalis*, *P. intermedia*, *Filifactor alocis*, *Tannerella forsythia*, *Parvimonas micra*, and *Fretibacterium* spp. (Abusleme et al., 2021). In another study, periodontitis was associated with an elevated number of pathogens such as *Parvimonas*, *Fusobacterium*, *Prevotella* as well as *F. alocis*, *P. gingivalis*, *T. forsythia*, and *Treponema denticola* (Abusleme et al., 2013; Dabdoub et al., 2016).

### 6.3 Oral cancer

Chronic inflammation caused by microorganisms in the oral cavity could potentially lead to the development of oral cancer by inhibiting cellular apoptotic processes and producing carcinogenic metabolites (Zhang et al., 2018). Anaerobic bacteria such as *Prevotella* spp., *Porphyromonas* spp. and *Fusobacterium* spp. can produce inflammatory cytokines such as interleukin-6 (IL-6), IL-1β, matrix metalloproteinases MMP-8 and MMP-9 and tumor necrosis factor-α (TNF-α) in response to LPS from Gram-negative bacteria, leading to chronic inflammation that is associated with invasive and aggressive tumor phenotypes, and epithelial tumor migration (Sun et al., 2020; Tuominen and Rautava, 2021). *Porphyromonas* spp. stimulates anti-apoptotic effects on tumor cells by reducing the tumor suppressor P53, activating the anti-apoptotic Jak1/Act/Stat3 signaling pathway and secreting an anti-apoptotic enzyme (nucleoside diphosphate kinase) (Karpiński, 2019).

Long-term periodontal disease is a major contributor to oral squamous cell carcinoma (OSCC). A decrease in Firmicutes and an increase in bacteria such as *Fusobacterium* spp., *Porphyromonas* spp., *Haemophilus* spp., *Peptostreptococcus* spp., *Rothia* spp., *Prevotella* spp., *Streptococcus* spp., and *Veillonella* spp. have been observed in OSCC (Lee et al., 2017; Yang et al., 2018). Furthermore, an increase in bacteria such as *Capnocytophaga leadbetteri*, *P. intermedia*, *Aggregatibacter segnis*, *Peptostreptococcus stomatis*, and *F. nucleatum* have been proved in the sites of buccal mucosal tumors in OSCC patients compared with normal tissue (Zhang L. et al., 2020). Oral microbiota can induce reactive oxygen species (ROS) and organic acids that may be involved in the development of cancer. Some species in the oral cavity, including S. oralis, *S. sanguinis*, *S. gordonii*, *Lactobacillus fermentum*, *L. minutus* and *Bifidobacterium adolescentis*, produce hydrogen peroxide (H2O2) through increased expression of NADPH oxidase activity *oxidase activity* (Abranches et al., 2018). In addition, *Alloprevotella* spp., *P. gingivalis*, *A. actinomycetemcomitans*, *P. intermedia*, *Peptostreptococcus* spp., and *F. nucleatum* produce free radicals such as hydrogen Sulfide, dimethyl Sulfide and methyl mercaptan, which affect tumor angiogenesis and growth (Hampelska et al., 2020; Wu et al., 2022).

Certain bacteria such as *Streptococcus* spp., *Lactococcus* spp., *Bifidobacterium* spp., *Lactobacillus* spp., *Pediococcus* spp. and *Leuconostoc* spp. produce lactic acid and lower the pH, which may provide an acidic microenvironment in tumor cells and increase the risk of metastasis (Vinasco et al., 2019).

## 7 Oral microbiota metabolites in oral disorders

There has been limited investigation into the characterization of oral microbiota metabolic products and their contribution to maintaining the balance between different types of bacteria and the human host in the oral microbiota (Ge et al., 2015) (Table 1). SCFAs are the best studied bacterial metabolites derived from the fermentation of carbohydrates and amino acids. SCFAs, including butyric, lactic, formic, acetic, and propionic acids, are necessary for reconstituting biofilm species and inducing tissue inflammation in the subgingival and supragingival oral cavity (Takahashi et al., 2010). Changes in the levels of individual metabolites due to changes in environmental conditions can lead to periodontal disease (Gomez-Arango et al., 2017).

*Streptococcus* and *Lactobacillus* are the major lactic acid producing bacteria in the oral cavity. The high levels of lactic acid in animal models of active caries indicate their important role in the cariogenic process (Olson et al., 2011). Lactic acid production by *S. gordonii* increases the pathogenicity of another oral microbiota such as *A. actinomycetemcomitans*. In addition, *Eubacterium* spp., *Veillonella* spp., and *Corynebacterium* spp. can catabolize lactic acid produced by *Streptococcus* spp. as a preferred substrate in oral biofilms (Zhang L. et al., 2020). A number of oral microbiota, including *P. intermedia*, *P. gingivalis*, *T. forsythia*, *F. nucleatum*, and *T. denticola* produce significant amounts of butyric acid (Mai et al., 2017). This metabolite, through an anti-inflammatory process, prevents an appropriate immune response against the bacteria involved in periodontitis and thus promotes the development of periodontal disease in the oral cavity (Lourenço et al., 2014). Butyrate is necessary in immune processes associated with periodontal disease by reducing the expression of the adhesion protein intercellular adhesion molecule-1 (ICAM-1) and the transmigration of immune cells, as well as reducing mediator production and neutrophil phagocytosis (Zhou et al., 2018). Butyrate accumulation can also stimulate the colonization of other oral microbiota such as *Actinomyces oris* in the early stages of biofilm colonization (Liu et al., 2021). It appears that SCFAs are also associated with cancer development. The acidic environment created by the acidogenic oral microbiota establishes an ideal environment for the growth and invasion of cancer cells (Abusleme et al., 2013). In addition, a number of SCFAs, including propionic, butyric, isobutyric, and isovaleric acids, can reactivate latent viruses such as Kaposi’s sarcoma-associated herpesvirus and Epstein–Barr virus to initiate tumorigenic processes in host cells (Reddy et al., 2019; Di Spirito et al., 2022). On the other hand, protein metabolism by the oral microbiota occurs in subgingival bacterial niches and deep periodontal pockets where access to dietary carbohydrates is limited (Gutt et al., 2018). The amino acid metabolizing bacteria *Fusobacterium*, produces alkaline by-products that raise the pH of the oral cavity to near neutral. This alkaline environment favors the colonization and activation of the proteolytic capacity of acid-sensitive oral bacteria such as *P. gingivalis*, which contributes to the initiation of inflammatory responses (Jin Baek et al., 2017). Some oral microbiota such as *P. intermedia* and *P. gingivalis* can metabolize acidic amino acids such as glutamic acid and aspartic acid to produce propionic acid and acetic acid. Increased metabolism of these amino acids is associated with the progression of periodontal pathology, particularly during pregnancy (Beck et al., 2020). In addition, *Bifidobacterium* spp. and *Scardovia wiggsiae* can synthesize acetic acid through the metabolic pathway and mediate pathogenicity through this by-product (Barbour et al., 2022). The production of bacterial metabolites in some cases has beneficial effects in oral diseases. Catabolism of the aromatic amino acid phenylalanine by redox reactions produces alkaline metabolites such as phenylacetate and regulates the pH of the oral biofilm (Roslund et al., 2021). The alkaline environment established by bacteria can neutralize the acidification of carbohydrate metabolism and stimulate the development of dental health by maintaining the microbiota in dental caries (Feng et al., 2023). In addition, alkali-producing species can balance acidic pH and reduce acidogenic bacteria, thereby inhibiting their tumor-promoting activities in oral cancer cells (Jiao et al., 2020). Some anaerobic oral microbiota, such as *P. intermedia*, *F. nucleatum*, and *P. gingivalis*, can metabolize the aromatic amino acid tryptophan to synthesize a variety of toxic indolic and phenolic metabolites, including indole, skatole, indole pyruvate, and indole acetate. These compounds, especially skatole, are odorous and contribute to oral halitosis (Zhang G. et al., 2020). *F. nucleatum* and *P. intermedia* may catabolize lipids and fatty acids in high-fat diets by lipases (Saha et al., 2023). Lipid metabolites may have significant pathogenic capacity. The metabolism of choline by *S. sanguinis* produces trimethylamine, which is converted to trimethylamine N-oxide in liver cell. High levels of trimethylamine N-oxide in the blood can interfere with reverse cholesterol transport and induce foam cell formation (Sánchez et al., 2014) (Table 2).

**Table 2:** Probiotics and their potential role in oral health.

	Bacteria	Mechanism	Outcomes	References
Probiotic	*Bifidobacterium* *Lactobacillus* *Streptococcus*		Increase alpha diversity in the oral microbiota Positive influence of additional probiotics Downregulate inflammatory processes Activate beneficial pathways such as type I and type II interferon responses	Willis and Gabaldon (2020)
	*Streptococcus* A12 *Streptococcus dentisani*	Arginine metabolism	Inhibition of acidic pH produced in cariogenic biofilms	Loozen et al. (2015), Huang et al. (2016), Lopez-Lopez et al. (2017), Willis and Gabaldon (2020)
	*Bdellovibrio bacteriovorus*	It invades the periplasmic space of Gram-negative bacteria by attacking the prey’s external surface and hydrolyzing its components, forming the bdelloplast	Treatment of periodontal disease	Bonfiglio et al. (2020)
	*L. rhamuosus* GG	Deplete glucose and repress ergosterol synthesis, inhibition of the growth of pathogens by fending off adhesion to oral epithelial cells	Prevent early caries in children	Nagaraj et al. (2012), Qiu et al. (2020)
	*Bifidobacterium* DN-173010, *L. rhamnosus* CG *L. reuteri* *L. casei*	Directly or indirectly alter gut microbiota through its metabolites, adhesion mechanisms, and gene regulation Reducing pro-inflammatory bacterial colonization and attenuating inflammatory responses	Change the colonizing of cariogenic bacteria Prevent dental caries	Busscher et al. (1999), Näse et al. (2001), Caglar et al. (2005), Caglar et al. (2006), Allaker and Ian Douglas (2015)
	*L. paracasei* GMNL-33	Inhibition the growth of many pathogenic microbes such as *S. mutans*	Reduction of *S. mutans* in saliva	Chuang et al. (2011)
	*L. gasseri* SBT2055	Improved expression of β-defensin	Prevention of *P. gingivalis* oral infection Reduction of IL-6 and TNF-ɑ mRNA levels in gingival tissue	Kobayashi et al. (2017)
	*L. lactis* NCC2211	Integrating into the oral biofilm	Prevent the growth of *Streptococcus sobrinus* OMZ176	Chuang et al. (2011), Mishra et al. (2020)
	*L. reuteri* RC-14 *L. rhamnosus* GR-1 *L. reuteri* ATCC PTA 5289	Prevent the connection of other pathogens by co-accumulation with yeasts	Inhibitory effect against *C. albicans* and *L. reuteri*	

## 8 Microbiota based therapies

### 8.1 Probiotics

Probiotics are live, non-pathogenic bacteria that provide health benefits to the host when consumed in adequate amounts. These microorganisms can alter the composition of the oral microbiota. The goal of probiotic therapy is to replace harmful indigenous microorganisms with non-pathogenic ones. Probiotics are often used to treat various oral and dental conditions, such as periodontal disease, halitosis, dental caries, and oral candidiasis (Bosch et al., 2012; Allaker and Ian Douglas, 2015; Mishra et al., 2020; Zhang Y. et al., 2022). Probiotics are available in various commercial forms, including tablets, toothpaste, and mouthwash, for the treatment of periodontal disease (Parcina Amizic et al., 2017; Zhang Y. et al., 2022). Systematic reviews by Inchingolo et al. (2023) and Navidifar et al. (2023) have shown the beneficial effects of probiotics on oral health conditions, including periodontitis, gingivitis, dental caries, and orthodontics. These bacteria interact directly with plaque, disrupt biofilm formation by competing for receptors on host tissue or other bacteria, and compete for nutrients. One significant mechanism involves the production of antimicrobial substances that inhibit oral microorganisms, such as organic acids, low molecular weight antimicrobial peptides, hydrogen peroxide, adhesion inhibitors, and bacteriocins. Probiotics also indirectly influence immune function by regulating both innate and adaptive responses, interacting with oral epithelial cells, and enhancing mucosal barrier function. Lactic acid bacteria can interact with immune cells like macrophages and T cells, leading to cytokine release and impacting systemic immunity (Lin T. H. et al., 2018; Mishra et al., 2020; Abdi and Ranjbar, 2022).

Commercial probiotics like *Bifidobacterium*, *Lactobacillus*, and *Streptococcus* have been shown to enhance alpha diversity in the oral microbiota (Willis and Gabaldon, 2020). Various theories suggest that probiotics such as *S. salivarius* strains can reduce inflammation and induce beneficial responses, including type I and type II interferon responses. *Streptococcus* A12 and *Streptococcus dentisani* can neutralize the acidic pH in cariogenic biofilms through arginine metabolism. For probiotics to exert their cariostatic effects, they must adhere to oral tissues and become part of the biofilm. Their effectiveness is also influenced by their duration of presence in the area. Dairy products like milk, yogurt, and cheese are effective carriers of probiotics. For instance, incorporating *Lactobacillus rhamnosus* GG into milk has been shown to help prevent early tooth decay in children (Nagaraj et al., 2012; Qiu et al., 2020). *Bifidobacterium* DN-173 010, *Lactobacillus rhamnosus* CG, *Lactobacillus reuteri*, and *Lactobacillus casei* have been demonstrated to disrupt cariogenic bacteria colonization, reducing the risk of dental caries (Busscher et al., 1999; Näse et al., 2001; Caglar et al., 2005, 2006; Allaker and Ian Douglas, 2015). A combination of live lyophilized acidophilic *lactobacilli* has been used topically to treat mild to moderate periodontitis (Lin T. H. et al., 2018). Coordination *Lactococcus lactis* NCC2211 into the verbal biofilm can avoid the development of *Streptococcus sobrinus* OMZ176. A biosurfactant inferred from *L. fermentum* that employments glucosyltransferases has been appeared to avoid *S. mutans* from creating sucrose, subsequently diminishing dental caries caused by this bacterium (Mishra et al., 2020). Considerations have appeared that *L. reuteri* RC-14, *L. rhamnosus* GR-1, and *L. reuteri* ATCC PTA 5289 can hinder the development of *C. albicans* by co-aggregating with yeasts, avoiding other pathogens from following to verbal surfaces (Chuang et al., 2011; Mishra et al., 2020).

*Bacillus subtilis* has a metabolic advantage over human oral pathogens, including *Streptococcus*. *B. subtilis* cells perform better in utilizing sugar alcohols like mannitol and sorbitol compared to *S. mutans*, which may explain the significant decrease in *S. mutans* biofilm production during co-culture (Duanis-Assaf et al., 2020). Reducing *S. mutans* could potentially help prevent caries from developing. Research indicates that gastric feeding of *Lactobacillus gasseri* enhances mice immunity against pathogenic bacteria by increasing bone resorption, periodontal ligament separation, and disruption, and reducing *P. gingivalis* (Kobayashi et al., 2017).

Chronic inflammation is a characteristic of cancer, and altered oral flora can lead to oral inflammation that promotes mutagenesis, angiogenesis, and uncontrolled cell proliferation, resulting in oral cancer (Kay et al., 2019; La Rosa et al., 2020). Anaerobic bacteria such as *Fusobacterium*, *Porphyromonas*, and *Prevotella* are common causes of the chronic inflammation associated with periodontal disease, and cause the release of inflammatory mediators (La Rosa et al., 2020; Vyhnalova et al., 2021; Bostanghadiri et al., 2023). Evidence suggests that probiotics help maintain oral health by selectively replacing and inhibiting dysbiosis. Postbiotics, including short-chain fatty acids (SCFA), polysaccharides, microbial cell fractions, functional proteins, EPS, and cell lysates, offer anticancer activity and can serve as an alternative to conventional chemotherapy (Żółkiewicz et al., 2020). Moreover, postbiotics of *Lactobacillus acidophilus*, *L. casei* and *Bifidobacterium longum* have shown significant anticancer effects on both implanted and chemically induced tumors. Administering the cytoplasmic fraction of lactic acid bacteria (LAB) *in vivo* enhanced specific antitumor activity by altering cellular immunity without causing direct tumor cell death (Lee et al., 2004). A study by Zhang et al. showed that probiotic *L. sali*var*ius REN* has the ability to inhibit oral cancer development in a dose-dependent manner through protection against oxidative damage and downregulation of COX-2 and proliferating cell nuclear antigen (Zhang et al., 2013). COX-2 is often up-regulated in many types of cancer, where it blocks apoptosis, alters cell adhesion, and manipulates the normal cell signaling pathway. Therefore, inhibiting COX-2 may be an effective strategy to prevent oral cancer by blocking the production of PGE 2, which promotes cancer growth (Mohan and Epstein, 2003). Also, Zhang et al. showed that oral gavage of *L. salivarius REN* or its postbiotic can potently inhibit the progression of 4-nitroquinoline-1-oxide-induced oral carcinogenesis by decomposing 4-nitroquinoline-1-oxide to a less toxic compound, preventing DNA from oxidative damage caused by the carcinogen, downregulating COX-2/PCNA expression, and inducing apoptosis. An *in vitro* study on human myeloid cells showed that probiotic *L. reuteri ATCC 6475* supernatant (Lr-S 6475) in combination with tumor necrosis factor (TNF) can induce apoptosis via NF-kB and MAPK pathways (Zhang et al., 2013). Secreted metabolites of *Acidobacter syzygii* exhibit anticancer effects by induction of apoptosis (Aghazadeh et al., 2017).

### 8.2 Microbiota transplantation

Growing antibiotic resistance has triggered interest in finding new treatments. Microbiota-centered interventions include a range of therapies that attempt to restore ecological balance by harnessing microorganisms (Bosch et al., 2012). Some examples of these interventions include microbiota transplantation, probiotics, engineered symbiotic bacteria, and microbiota-derived proteins and metabolites (Sorbara and Pamer, 2022). One of the alternative treatments for oral diseases is oral microbiota transplantation (OMT), a type of treatment that involves altering the dysbiosis of the microbiota to restore the microbial ecological balance.

OMT works by transferring biofilms from healthy donors to patients with caries or periodontitis. The proposed procedure, as hypothesized by Dewhirst and Hoffmann, involves three steps: (1) collecting supragingival plaque from a caries-free donor who may be a family member of the recipient patient, (2) preserving the plaque in saline solution, and (3) transferring the plaque sample to the teeth of a patient with active caries using a nylon swab (Pozhitkov et al., 2015; Mira et al., 2017). It is important to have a donor with a strong oral microbiome that is free of cariogenic bacteria, particularly *S. mutans*, and shows a slight decrease in pH when exposed to a sugar stimulus.

It is likely that interspecies interactions and immunostimulatory effects will play a significant role in OMT therapy. OMT reduced systemic inflammation induced by neck and head irradiation by reducing the levels of IL-1, IL-6, TNF-α, and TGF-β (Xiao et al., 2021). However, transplanted oral biofilms must be able to withstand selective environmental pressures and continue to exert beneficial effects. OMT is hypothesized to work by establishing colonization of oral sites, competing with pathogenic bacteria for adhesion sites and nutrient sources, producing bacteriocin and hydrogen peroxide to eliminate pathogens, and controlling local and systemic immune responses (Nascimento, 2017). The safety issues related to the use of OMT and oral probiotics are comparable. In similar to probiotics, transplanted biofilms must be disease-free and have impressive genetic stability. However, how OMT works and the best way to deliver it is not understood (Nath et al., 2021). As of now, it is uncertain if oral biofilms should be directly transplanted from a donor in good health into a recipient, or if they should be pre-treated to reduce the levels of pathogenic bacteria before transplantation. It is also uncertain whether biofilms produced *in vitro* or naturally occurring commensal organisms would be a superior alternative (Sánchez et al., 2014). Nevertheless, biofilms of clinical strains with useful and health-promoting properties are effective in interacting with and replacing the biofilms associated with the disease (Gutt et al., 2018).

Other factors to be considered include the need to disinfect the recipient’s oral cavity before OMT and whether single or several doses of oral transplants would be necessary for successful and sustained colonization of the oral cavity for the establishment and maintenance of health (Nascimento, 2017).

### 8.3 Antimicrobial agents targeting specific oral pathogens

The most common form of oral pathogen eradication is the use of antibiotics. Some systemic antibiotics that target specific oral pathogens include penicillin, metronidazole, macrolides, and clindamycin. Metronidazole is a topical medication that is effective against obligate anaerobic microorganisms in the oral cavity, including those from infected necrotic pulp. In *in vitro* experiments, metronidazole inhibited over 99% of the bacteria found in carious lesions and infected root dentin (Delaviz et al., 2019). Erythromycin is a macrolide commonly used to treat various bacterial infections. According to some evidence, caries-active dams can become caries-inactive when individuals receive erythromycin. In addition, the treatment with erythromycin resulted in a 35% reduction in plaque formation within one week (Qiu et al., 2020).

Penicillin has been shown to be effective against several strains of gram-positive *Streptococci* and *Staphylococci*, as well as certain gram-negative bacteria. However, bacterial resistance to β-lactam antibiotics is a major challenge (Macy, 2015). Over the years, the use of systemic antibiotics for the treatment of oral bacteria has decreased. This is because many of these antibiotics not being originally designed for this purpose, and they are lack of specificity in treating oral diseases. Instead, alternative antimicrobial agents have been introduced that specifically target oral bacteria, including chlorhexidine, fluoride, antimicrobial peptides (AMPs), and quaternary ammonium salts. Fluoride, the major anion of fluorine, has been demonstrated to be a very powerful agent in the prevention of tooth decay of dental caries, especially when added to consumed water or to oral products such as toothpastes, mouthwashes, and oral supplements. There is an ongoing debate about whether fluoride helps prevent tooth decay (O’Mullane et al., 2016). It is generally believed that the fluoride ions interact with the tooth’s mineral structure, thus leading to more remineralization and avoiding demineralization due to cariogenic bacteria’s acidity (Thurnheer and Belibasakis, 2018).

Chlorhexidine, a cationic polybiguanide, was an early antiseptic that was recommended for dental decay and has been demonstrated to be the most effective. It remains the most important antiplaque agent because of its ability to prevent plaque formation and bacterial adhesion. Disruption of acidic groups of glycoproteins in saliva by chlorhexidine reduces plaque adhesion. It also prevents bacterial adhesion to tooth surfaces, either by adhering to extracellular polysaccharides or by the competition with calcium ions for accumulation in plaque (Brookes et al., 2020).

Initially, quaternary ammonium salts were added to mouthwashes to combat oral plaque. Later, they have been added to dental products to provide antimicrobial properties paliguanid (Ge et al., 2015). AMPs are widely distributed in the human body and exhibit antimicrobial properties that target microorganisms (Gordon et al., 2005; Zasloff, 2019). AMPs are considered as one of effective molecules in the eradication of oral pathogens. They are derived from larger molecules and consist of a signal sequence along with various modifications such as glycosylation, proteolysis, amino acid isomerization (Khurshid et al., 2016). AMPs are classified into different categories based on their amino acid composition, size, and structural configurations (Brogden, 2005; Harris et al., 2009). Oral epithelial cells, salivary glands, and neutrophils contribute to the production of various AMPs in saliva, which together provide a powerful defense due to their antibacterial, antioxidant, and antifungal properties (Kutta et al., 2006; Güncü et al., 2015). The most common AMPs expressed in the oral cavity are including α-, and β- defensins (shown as hNP), histatins, statherin, adrenomedullin, azurocidin, and cathelicidins (LL37). The majority of hNP-1 to 3 is concentrated in saliva, gingival epithelia, tongue, palate, buccal mucosa, and salivary glands/ducts and accounting for approximately 99% in a healthy individual (Dale and Krisanaprakornkit, 2001). Dale et al. found that individuals with dental caries have reduced levels of α-defensins (hNP-1, -2, and -3) in their saliva, suggesting that these biomarkers could be used to assess caries risk in the general population (Dale et al., 2006). It has been proposed that hBD-1 is constitutively expressed and has the function of inhibiting the transformation of normal flora into harmful microbes, while hBD-2 and -3 are induced by bacterial lipopolysaccharides (LPS), proinflammatory mediators (Interleukins [IL-1β], tumor necrosis factors [TNF-α], interferons [IFN-γ]) and are more effective in the control of nearly all types of pathogens (Khurshid et al., 2016). Histatins, a group of low positively charged histidine-enriched peptides found in saliva, are produced by cells of the parotid and submandibular salivary ducts in healthy adults (De Sousa-Pereira et al., 2013). Histatins often have antifungal activity and are composed of three main members of His-1, His-3 and His-5 (Bikker, 2020). Their antifungal mechanism is exerted in several steps, including the inhibition of mitochondrial respiration by forming reactive oxygen species, entering the cell by mobilizing ions (K+, Mg2+) that results in cell death (Khurshid et al., 2016). For example, Hst-5 is used in a mouthwash (Demegen) for oral candidiasis in patients with human immunodeficiency virus HIV because of its potent limiting effects on Candida biofilm (Pusateri et al., 2009; Gorr, 2012). In humans, the oral cavity and respiratory tract have a single representative of cathelicidins called human cationic antimicrobial peptide (hCAP18) (Murakami et al., 2002). Numerous studies have shown that LL37/hCAP18 is effective against a wide range of microorganisms, including gram-negative and gram-positive bacteria, fungi, viruses, and parasites (López-García et al., 2005; Ridyard and Overhage, 2021; Aloul et al., 2022; Bhattacharjya et al., 2024). It rapidly eliminates bacteria by creating ionic channels in their cell walls and by its ability to bind to LPS in gram-negative bacteria (Sancho-Vaello et al., 2020). Another study demonstrated the enhanced bactericidal activity of synthetic peptides derived from LL37/hCAP18 against Streptococcus sanguis (Isogai et al., 2003). In addition, Ouhara et al. demonstrated a higher activity of LL37 against a group of oral bacteria including *S. mutans*, *S. sanguinis*, *S. sali*var*ius* and *S. mitis* when compared to human b-defensins (Ouhara et al., 2005). Adrenomedullin is a disulfide bonded cationic amphipathic antimicrobial peptide found in high concentrations in whole saliva. Adrenomedullin levels are significantly elevated in periodontally compromised regions compared to healthy regions (Kapas et al., 2004; Gorr, 2009). Statherin is a basic histidine-rich peptide of the histatin/statherin family whose antimicrobial properties are observed in its C-terminus (Wilmarth et al., 2004). Statherin is found in saliva and gingival crevicular fluid and inhibits the growth of anaerobic bacteria of the oral cavity (Denny et al., 2008). Azurocidin, a protein found in saliva, exhibits potent antibacterial activity against Gram-negative bacteria by binding strongly to lipopolysaccharide (Dhaifalah et al., 2014). Essential oils (EOs) also are one of other options in treating infection associated with oral cavity. EOs have been the focus of research for many years as effective antimicrobial agents and are applied in various medical fields, including dentistry (Radu et al., 2023). EOs derived from aromatic plants, are known for their strong odor and complex nature, containing hundreds of elements with a predominance of two or three major components. Hydrocarbon terpenes and terpenoids form the main components, with the majority of terpenoids being monoterpenes and sesquiterpenes, and the remaining group being oxygenated derivatives of hydrocarbon terpenes (Elyemni et al., 2019). Monoterpenes have been extensively researched for their potential to treat a number of diseases. Terpenes and terpenoids, along with aromatic and aliphatic elements, are responsible for the bactericidal or bacteriostatic effects of EOs (Elyemni et al., 2019; Machado et al., 2022). In addition, the antimicrobial activity of EOs depends on their unique composition, structure, amount and potential interactions (Zomorodian et al., 2015; Jafri and Ahmad, 2020). EOs have been shown to have antibacterial, antifungal, and anti-inflammatory properties that target oral pathogens (Karadağlıoğlu et al., 2019). The mechanism of action of EOs is primarily by increasing the permeability of cell membranes, leading to the leakage of ions and cellular components, ultimately causing cell lysis (Karan, 2019). EOs can also interfere with protein synthesis or cell division through stimulating the production of reactive oxygen species, leading to cell death (Lakhdar et al., 2012). Toscano-Garibay et al. indicated that among the oral pathogens tested, *S. mutans* is the most susceptible to EOs, followed by *S. sobrinus*, *S. salivarius*, *S. sanguis*, and *L. acidophilus* (Toscano-Garibay et al., 2017). There are limited studies on the efficacy of EOs against *C. albicans*. Oregano oil was found to inhibit the attachment and development of *C. albicans* biofilm and to reduce biofilm formation on surfaces pretreated with this oil (Karan, 2019). The results of a clinical study showed that regular use of an EO-based mouthrinse significantly reduced plaque, gingivitis, and periodontitis compared to a 0.05% cetylpyridinium chloride mouthrinse (Dagli and Dagli, 2014). Essential oils have the potential to inhibit plaque, providing additional protection to soft tissues from bacterial damage. While chlorhexidine (CHX) is often preferred for plaque control and the treatment of gingivitis and periodontitis, EOs are considered the most reliable substitute (Dosoky and Setzer, 2018; Kajjari et al., 2022). Sweet orange, bitter orange, lemon, lime, grapefruit, bergamot, yuzu and kumquat have been shown to have medicinal properties when used in mouthwash (Radu et al., 2023). *E. faecalis* is frequently present in root canals identified with apical periodontitis and is a main cause of secondary endodontic infections (Reiznautt et al., 2021). According to a study by Gokalp et al., a combination of calcium hydroxide and two EOs (*M. spicata* and *O. dubium*) showed significant antimicrobial effects (Nabavizade et al., 2018).

## 9 Conclusion and perspectives

As our understanding of the oral microbiota advances, it is becoming increasingly clear that maintaining a balanced microbial community is critical for optimal oral health. Disruption of this balance can lead to the proliferation of pathogenic bacteria, resulting in conditions such as dental caries and periodontitis. Therefore, a comprehensive approach to oral health must include both preventive and therapeutic strategies. Preventive measures, including good oral hygiene, a balanced diet and the avoidance of risk factors, are essential. However, due to the limitations and temporary efficacy of conventional treatments, there is a growing need for innovative solutions. Probiotics and microbiota transplantation are promising ways to maintain the microbial balance. Probiotics, available in various commercial formulations, have shown considerable success, while microbiota transplantation, although relatively new, offers a compelling area for further investigation. As we continue to elucidate the complex interactions between the oral microbiota and the host, it is imperative that we continually re-evaluate and improve our preventive and therapeutic approaches to ensure long-term oral health.
